# 

**Published:** 1938

**Authors:** 


### The Medical Library of the University of Bristol

WITH WHICH IS INCORPORATED

**The Library of the Bristol Medico-Chirurgical Society.**

---

*The following donations have been received since the publication of the list in October,* 1938.

December, 1938.

| | |
|---|---|
| Dr. E. G. C. F. Atchley (1) .. .. .. | 3 volumes. |
| C. T. Campion Bequest (2) .. .. .. | 1 volume. |
| Carnegie Institution (3) .. .. .. | 2 volumes. |
| Professor E. W. Hey Groves (4) .. .. | 8 volumes. |
| Sir W. Arbuthnot Lane (5) .. .. .. | 1 volume. |
| W. R. Lefanu (6) .. .. .. .. | 1 volume. |
| National Association for the Prevention of Tuberculosis (7) .. .. .. .. | 1 volume. |
| Professor C. Bruce Perry (8) .. .. | 1 volume. |
| Royal College of Surgeons (9) .. .. | 1 volume. |

Unbound periodicals have also been received from Dr. R. F. Barbour, Professor E. W. Hey Groves, Professor C. Bruce Perry, Dr. G. R. A. de M. Rudolf and Dr. J. Odery Symes.

### THE ONE HUNDRED AND SIXTY-FIFTH LIST OF BOOKS

The figures in round brackets refer to the figures after the names of the donors. The books to which no such figures have been attached have either been bought from the Library Fund or received through the *Journal*.

**Bardswell, N. D.** .. *Tuberculosis in Cyprus* .. .. .. .. .. (7) 1937

**Benedict, F. G., and Lee, R. C.** *Hibernation and Marmot Physiology* .. .. .. .. .. .. .. .. (3) 1938

**Bircher, E.**.. .. .. *Das Kropfproblem* .. .. .. .. .. .. .. (4) 1937

**Bramwell, C., and Longson, E. A.** *Heart Disease and Pregnancy* .. 1938

**Buckley, C. W.**.. .. *Arthritis, Fibrositis and Gout* .. .. .. .. 1938

*Catalogue of Lewis's Medical and Scientific Lending Library.* Part I. Authors and Titles .. .. .. 1938

**Chamberlain, E. Noble.** *Symptoms and Signs in Clinical Medicine* 2nd Ed. 1938

**Darling, H. C. R.** .. *Surgical Nursing and After-Treatment* 6th Ed. (4) 1938

**Drury, E. G. Dru** .. *Psyche and the Physiologists* .. .. .. .. .. 1938

**Elliott, John** .. .. *The Medical Pocket-book* .. .. 3rd Ed. (1) 1791

**Ewart, E. D.** .. .. *A Guide to Anatomy* .. .. .. 4th Ed. 1938

**Fox, R. Fortescue** .. *Evolution of Chronic Rheumatism* .. .. 1938

**Freind, John** .. .. *Chymical Lectures* .. .. .. .. .. .. .. 1712

**Friedländer, E.** .. .. *Die Therapie der Thrombose* .. .. .. (4) 1938

**Harris, K., and Harris, E.** *Minor Medical Operations* .. .. .. 1938

**Henry, Sir E. R., Bart.** *Classification and Uses of Finger Prints* .. 1937

**Hirschfeld and Leveillé** *Neurologie ou Description et Iconographie du Système Nerveux* .. .. .. .. .. .. (1) 1853

**Hobson, F. G.** .. .. *Medical Practice in Residential Schools* .. 1938

**Hodges, N.** .. .. *Loimologia ; or an Historical Account of the Plague in London in* 1665 .. 3rd Ed. (1) 1721

**Jamieson, E. B.** .. *Illustrations of Anatomy for Nurses* .. (4) 1938

**Janssen, P.** .. .. *Chirurgische Erkrankungen der Harnorgane* (4) 1938

**Joules, H.** .. .. .. *The Doctor's View of War* .. .. .. .. 1938

**Kress, H. F., and Kittler, W.** *Innere Medizin in der Chirurgie* (4) 1938

**Laignel-Lavastine, M., and Koressios, N. T.** *Recherches sur la Sclérose en Plaques* .. .. .. .. .. .. .. (8) 1938

**Lane, Sir W. Arbuthnot** *Bibliography of Published Writings* .. (5) 1938

**Le Fanu, W. R.** .. *British Periodicals of Medicine* .. .. (6) 1938

**Micks, R. H.** .. .. *Essentials of Materia Medica, Pharmacology and Therapeutics* .. .. .. 2nd Ed. 1938

**Møller-Christensen, V.** *The History of the Forceps* .. .. .. .. 1938

**Nixon, J. A., and Nixon, D. G. C.** *Text-book of Nutrition* .. .. .. 1938

**Shattuck, G. C.** .. *A Medical Survey of the Province of Guatemala* (3) 1938

**Still, G. F.** .. .. *Common Happenings in Childhood* .. .. 1938

*Treatment in General Practice.* Articles reprinted from *The British Medical Journal.* Vols. I–II .. 2nd Ed. 1938

**Vaubel, E.** .. .. *Die Arthroskopie* .. .. .. .. .. .. (4) 1938

**Watson, J. K.** .. .. *Anaesthesia and Analgesia for Nurses and Midwives* .. .. .. .. .. .. .. .. .. .. 1938

**Weeks, C. C.** .. .. *Alcohol and Human Life* .. .. 2nd Ed. 1938

**Wells, C.** .. .. .. *A Manual of Surgery for Nurses* .. (4) 1938

### TRANSACTIONS, REPORTS, JOURNALS, ETC.

Calendar of the Royal College of Surgeons, 1938 .. .. .. (9) 1938

Historisch-literarisches Jahrbuch für die deutsche Medicin. Vols. I–III 1838–40

Medical Directory, 1939 .. .. .. .. .. .. .. .. .. .. .. .. 1938

Natural Food. Vols. I–III .. .. .. .. .. .. .. .. (2) 1891–93

Quarterly Cumulative Index Medicus. Vol. 23 .. .. .. .. .. 1938

Reports on Chronic Rheumatic Diseases. No. 4 .. .. .. .. 1938

St. Bartholomew's Hospital Reports. Vol. LXXI .. .. .. .. 1938

### Publications Received

From Messrs. Baillière, Tindall & Cox :

*The Health of the Nation and Deficiency Diseases.* By John Maberly, M.R.C.S.(Eng)., L.R.C.P.(Lond.). Price 5s.

*Whitla's Dictionary of Treatment.* 8th Edition. By R. S. Allison, M.D., M.R.C.P.(Lond.), and C. A. Calvert, M.B., B.Ch., F.R.C.S.I. Price 30s.

From Messrs. Bayer Products Ltd. :

*Fifty Years of "Bayer" Remedies.* 1888–1938.

From Messrs. Cassel & Co. Ltd. :

*A Handbook of Midwifery.* By Sir Comyns Berkeley, M.A., M.C., M.D.(Cantab.), F.R.C.P.(Lond.), F.R.C.S.(Eng.), M.M.S.A., F.C.O.G. 10th Edition. Price 8s.

From Messrs. Eyre & Spottiswoode Ltd. :

*Modern Anæsthetic Practice.* Edited by Sir Humphry Rolleston, Bart., G.C.V.O., K.C.B., M.D., F.R.C.P., and Alan A. Moncrieff, M.D., F.R.C.P. Price 10s. 6d.

From Messrs. H. K. Lewis & Co. Ltd. :

*Catalogue of Lewis's Medical and Scientific Lending Library.* Part I. Authors and Titles. New edition, revised to the end of 1937. Price 16s. (to subscribers 8s.).

*Cardiovascular Disease in General Practice,* By Terence East, M.A., D.M., F.R.C.P. Price 10s. 6d.

*Handbook of Sanitary Law for the use of Candidates for Public Health Qualifications.* By B. Burnett Ham, M.D., D.P.H. 12th Edition. Price 7s. 6d.

*Minor Medical Operations.* By Kenneth Harris, M.A., M.D., F.R.C.P., and Edith Harris, M.B., B.S., D.P.H. Price 7s. 6d.

*More Thoughts of a Doctor.* By F. Parkes Weber, M.A., M.D., F.R.C.P., F.S.A. Price 1s. 6d.

*Alcohol and Human Life.* By Courtenay C. Weeks, M.R.C.S., L.R.C.P. Price 6s.

From Oxford University Press (Humphrey Milford) :

*A Cerebral Atlas.* By Richard J. A. Berry, M.D., F.R.S.E., F.R.C.S.E. Price £5 5s.

*Iodine Metabolism and Thyroid Function.* By A. W. Elmer, M.D. Price 30s.

*Oxygen and Carbon-dioxide Therapy.* By Argyll Campbell, M.D., D.Sc.(Edin.), and E. P. Poulton, M.A., D.M.(Oxon.), F.R.C.P.(Lond.). 2nd Edition. Price 12s. 6d.

*Physiology of the Nervous System.* By J. F. Fulton, M.A., D.Phil., S.B., M.D. Price 25s.

*Medical Practice in Residential Schools.* By F. G. Hobson, D.S.O., D.M., F.R.C.P. Price 10s. 6d.

From MESSRS. JOHN MURRAY :

*St. Bartholomew's Hospital Reports.* LXXI. Price 15s.

From MESSRS. JOHN WRIGHT & SONS LTD :

*British Journal of Surgery.* Vol. XXVI. No. 102. Price 12s. 6d.

*Symptoms and Signs in Clinical Medicine.* By E. NOBLE CHAMBERLAIN, M.D., M.Sc., F.R.C.P. 2nd Edition. Price 25s.

*Imperial and Metric Conversion Scales.* By J. W. THORNTON, M.A., B.M., M.R.C.P. Price 6s.

### Local Medical Notes

EXAMINATION RESULTS.

### University of Bristol.

M.D. (by Thesis).—C. H. G. Price.

M.B., Ch.B.—*Second Examination. Pass :* A. J. F. Saxton, Dorothy Hughes, W. G. H. Hunt, Mary Jacob, Mary E. Loewe, Jyotikana Datta.

M.B., Ch.B. (Section I).—*Final Examination. Pass :* Ruth Appleby,† Dorothy M. Ayre, D. L. Bayley, D. C. Bodenham,† Jean A. Butt, Mary Crago, J. N. P. Davies,† Marjorie O. Dunster,† J. L. Elliott,† J. L. Emery, Sara M. Field-Richards, E. M. Grace, Betty F. Hannaford,† F. R. Hurford, Rosemary W. Knowles, N. E. Melling, C. A. St. Hill,†‡ Jeannette Shed,† Dorothy M. Shotton,† P. R. H. Slade, A. A. Stonehill, E. M. Wagstaff,† T. H. White.†

B.D.S.—*Third Examination. Pass : In Dental Mechanics and the Properties of Materials used in Dental Practice :* F. C. Baker, J. R. D. Gordon.

B.D.S.—*Final Examination. Pass :* G. R. Styles.

L.D.S.—*Second Examination. Pass : In Dental Anatomy :* B. N. Neilson. *In Anatomy and Physiology :* L. Murray, R. S. Saxton.

† With distinction in Materia Medica, Pharmacy, Pharmacology, Pharmacotherapeutics and Toxicology.

‡ With distinction in Pathology and Bacteriology.

L.D.S.—*Third Examination. Pass: In Dental Mechanics and the Properties of Materials used in Dental Practice:* J. R. Herbert, O. H. Minton, C. R. Sage, R. H. G. Sims. *In Dental Materia Medica:* J. C. Jowett, R. B. Stiles.

L.D.S.—*Final Examination. Pass:* *A. J. H. Orchard, *Peggy M. Rooke, F. W. Clift, J. W. S. Jenkins.

**Conjoint Board.**—L.R.C.P., M.R.C.S.—*Pass:* Margot H. Dickson (Anatomy), D. J. Jones (Anatomy and Physiology), *Margaret E. M. Slater (Medicine), R. T. Moore (Medicine), E. A. Griffiths (Pathology and Midwifery), A. L. T. Beddoe (Midwifery), E. B. Hughes (Pathology), Rotha H. Barnfield (Surgery), Joan E. Mackworth (Surgery), E. Meriel Wagstaff (Pathology).

Diploma in Child Health.—*Pass:* R. G. Wilbond.

* Qualified.

---

### NOTICE TO CONTRIBUTORS.

Illustrations should always be supplied ready for reproduction. All re-touching or other operations required will be charged to the Author. Line drawings should be drawn in Indian ink.

Authors of original articles are entitled to receive twenty-five Reprints, with covers showing source of Reprint free of charge. Additional Reprints may be obtained at the following rates (*provided notice is given at the time of returning proofs*):—

**ADDITIONAL COPIES.**

| | | | | | |
|---|---|---|---|---|---|
| (*a*) With cover showing source of Reprint (no name or title) .. | 25 copies, | 8 pages, | **5/6** | 16 pages, | **9/6** |
| | 50 " | " | **8/6** | " | **16/-** |
| (*b*) With special cover (showing source of Reprint, Author's name and title of paper) .. .. .. | 25 " | " | **13/-** | " | **17/-** |
| | 50 " | " | **18/-** | " | **25/6** |

**Illustrations.**—For 25 copies of one page, **9d.** extra. For 50 copies of one page, **1/6** extra.

NOTE.—Contributors requiring special Covers on their free Reprints may obtain same at the following rates:—

| | | |
|---|---|---|
| Cover showing source of Reprint and Author's name and title of article .. .. .. | 25 copies, **7/6** | 50 copies, **9/6** |

### Index

Abreu, A. L. d'.—Thoracic surgery, 43

Adams, A. W.—Lobectomy for bronchiectasis, 63

Air as a conductor of electricity.—A. M. Tyndall, 211

Appointments:—

Miss Helen Bell, Matron at Bristol Royal Infirmary, 150

Miss A. Cameron, Matron at St. Monica's Home of Rest, 150

T. F. Hewer, Professor of Pathology in the University of Bristol, 141

Association of Physicians, 141

Berry, R. J. A.—Cerebral malformations, 111

Blind, A guide dog for the.—F. W. Willway, 235

Book Reviews, 67, 132, 199, 245

Bristol, Standard of Living in, 260

Bronchiectasis, Lobectomy for.—A. W. Adams, 63

Burnham-on-Sea, Medicinal waters.—J. A. Nixon, 191

Cerebral malformations.—R. J. A. Berry, 111

Chitty, H. — Some tuberculous conditions, 161

Colston Research Society, Social Survey, 260

Daviesville.—J. A. Nixon, 191

Devis, Harry Francois.—Obituary, 266

Drinker's "Iron Lung" and other artificial respirators.—J. A. Nixon, 239

Electricity, Air as a conductor of.—A. M. Tyndall, 211

Eskell, Neil Patrick.—Obituary, 266.

Examination Results, 91, 149, 210, 273

Foss G. L.—Clinical applications of the male and female sex hormones, 23

Gee, C. A. H.—Obituary, 145

Hewer, T. F.—Syphilis in the tropics, 217

Insulin, Protamine.—J. A. Nixon, 121

Intra-cranial tumours.—F. W. Willway, 151

Library, 86, 146, 207, 270

Library Association, University and Research Section, 203

Ling, T. M.—Psychological factors in disease, 101

Lobectomy for bronchiectasis.—A. W. Adams, 63

Local Medical Notes, 91, 149, 210, 273

Long Fox Memorial Lecture.—A. M. Tyndall, 211

MacWatters, J. C.—Obituary, 83

Medical defence.—C. A. Moore, 1

Moore, C. A.—Medical defence, 1

Nixon, J. A.—Use of protamine insulin, 121

Nixon, J. A.—Medicinal waters at Burnham-on-Sea, 191

Nixon. J. A.—Drinker's "Iron Lung" and other artificial respirators, 239.

Nursing (Editorial), 80

Obituaries—

Harry Francois Devis, 266

Neil Patrick Eskell, 266

Obituaries—(*continued*)
C. A. H. Gee, 145
J. C. MacWatters, 83
Patrick Watson-Williams, 262
C. G. Russ Wood, 205
Hartland S. Wright, 82

Presidential Address.—C. A. Moore, 1
Protamine insulin, Use of.—J. A. Nixon, 121
Psychological factors in disease.—T. M. Ling, 101
Publications received, 90, 148, 209, 272

Reviews of Books, 67, 132, 199, 245
Royal Fort, History of, 142

Societies :—
Bristol Medico-Chirurgical, 85, 146, 267
Sex hormones, Clinical application of the male and female.—G. L. Foss, 23
Sinclair, H. M.—Vitamin B complex, 173
Syphilis in the tropics.—T. F. Hewer, 217

Thoracic surgery.—A. L. d'Abreu, 43
Development of (Editorial), 78
Tuberculous conditions, Some.—H. Chitty, 161
Tumours, Intra-cranial.—F. W. Willway, 151
Tyndall, A. M.—Air as a conductor of electricity (Long Fox Memorial Lecture), 211
The Standard of Living in Bristol (Editorial), 260

Vitamin B complex.—H. M. Sinclair, 173

Watson-Williams, P.—Obituary, 262
Willway, F. W.—A guide dog for the blind, 235
Intra-cranial tumours, 151
Wood, C. G. Russ.—Obituary, 205
Wright, H. S.—Obituary, 82

